# Impact of Deep Cryogenic Treatment on X210CrW12 Steel’s Wear Behavior and Microstructural Characteristics

**DOI:** 10.3390/ma18040879

**Published:** 2025-02-17

**Authors:** Onur Özbek, Nursel Altan Özbek

**Affiliations:** 1 Department of Machinery and Metal Technologies, Gümüşova Vocational School, Düzce University, Düzce 81850, Türkiye; onurozbek@duzce.edu.tr; 2 Department of Machinery and Metal Technologies, Dr. Engin Pak Cumayeri Vocational School, Düzce University, Düzce 81700, Türkiye

**Keywords:** X210CrW12 steel, deep cryogenic treatment, holding time, microstructure, hardness, wear resistance

## Abstract

In this work, the microstructure, hardness, tensile and yield strength, and wear resistance of X210CrW12 steel are examined in relation to the impacts of deep cryogenic treatment. In order to achieve this, deep cryogenic treatment at −180 °C was applied to X210CrW12 steel samples for 12, 18, 24, and 36 h following quenching. Following the cryogenic treatments, a tempering heat treatment was applied to the samples at 300 °C for two hours. Experimental results showed that deep cryogenic treatment significantly improved the mechanical properties of X210CrW12 steel. The best mechanical properties were obtained after applying deep cryogenic treatment for 24 and 36 h. The cryogenic treatment showed the most dominant effect on wear resistance. As a result of the wear tests performed with the pin-on-disk method, it was determined that the wear rate of the sample subjected to cryogenic treatment for 36 h was 59.37% less than that of the conventionally hardened sample. The deep cryogenic treatment applied for 36 h provided the highest hardness increase of 12.06%. Compared to the conventionally hardened sample, the tensile and yield strength in the steel subjected to deep cryogenic treatment for 24 h is up to 11.02% and 11.35% higher. As a result of microstructural analysis, it was determined that it provides cryogenic new carbide precipitation, increases carbide density, and provides a more homogeneous carbide distribution.

## 1. Introduction

Cryogenic treatment is a heat treatment process that involves cooling the material and is applied to improve the mechanical properties of materials, complementing traditional heat treatment. Samples undergoing deep cryogenic treatment are progressively cooled to cryogenic temperatures between −125 °C and −196 °C, maintained there for predetermined amounts of time, and then gradually warmed back up to the surrounding temperature. In this approach, the materials’ mechanical characteristics are enhanced and austenite is transformed into martensite [1], fine carbide particles are formed, and the carbide particles are distributed uniformly [2,3]. It is possible to come across two different theories in the literature to explain carbide precipitations. The first of these can be explained as the carbon atoms jumping from the martensite matrix to dislocations, twinning, and other defects as a result of the contraction of the martensite matrix during deep cryogenic treatment and the formation of new carbide nucleation regions in these regions during the tempering process. In addition, this situation causes the tetragonality of the body-centered tetragonal structure, which is the crystal lattice structure of martensite, to decrease, and thus the internal stresses of the material decrease [4,5]. The second theory can be explained as the formation of carbides in the dislocation gaps due to the dislocation movements in the austenite and martensite structures. The dislocation mobility in the internal structure occurs as a result of the transformation of austenite to martensite and the local plastic deformation that occurs due to the volume difference resulting from this phase transformation. Plastic deformation forces the dislocations to move, bringing the carbon atoms together in the dislocation gaps. The carbon atoms in the dislocation gaps form nucleation regions for the hard carbide structures before tempering [5,6]. Cryogenic treatment significantly influences the characteristics of freshly formed martensite, which, when tempering, precipitates carbide particles, consequently increasing both toughness and wear resistance. Improved wear resistance in steels is due to the precipitation of fine carbides during cryogenic treatment [4]. The formation of high carbide contraction will increase wear resistance, reduce friction, and improves resistance [4,7]. A more homogeneous carbide distribution can be achieved in the microstructure of tool steels due to the secondary carbides precipitated after cryogenic treatment and tempering [5,7].

Cryogenic treatment has different application conditions, such as cryogenic treatment holding temperature, holding time, and cooling rate. Chen et al. [8] reported in their study using 51CrV4 spring steel that, considering the overall performance in hardness, impact toughness, and wear resistance, cryogenic treatment holding time had the most significant effect on performance, with a contribution rate of 49.01%. They found that this was followed by cooling rate, with 27.74%, the next most prominent effect was the number of cryogenic treatment cycles, with 18.85%, and the lowest effect was the cryogenic treatment temperature, with a rate of less than 5%. In another study, Darwin et al. [9] optimized the cryogenic treatment parameters applied on SR34 martensitic stainless steel using the Taguchi method to obtain maximum wear resistance. As a result of the study, they showed that the most important cryogenic treatment parameter is the holding temperature, with a rate of 72%, the second most important factor is the holding time, with a rate of 24%, and the third important factor is the cooling rate, with a rate of 10%. The wear resistance and hardness value of AISI D5 steel that had been cryogenically treated and untreated were examined by Yan et al. [10]. The results were interpreted as follows: cryogenic treatment increases hardness and wear resistance by reducing residual austenite. Tool steels of types M390 and M398 made using powder metallurgy–HIP techniques were also the subject of Studeny et al. [11]. Compared to the M390 material, the M398 material demonstrated a wear reduction of almost 400%. A cryogenic quench to −78 °C and a tempering temperature of 400 °C made up the optimal HT. Deep cryogenic heat treatment considerably decreased residual austenite in AISI D2 cold tool steels, and secondary carbides exhibited precipitation behavior, according to Das et al.’s study [12]. With cryogenic heat treatment, it was found that the hardness rose. They concluded that cryogenic heat treatment greatly enhanced AISI D2 cold tool steel’s wear resistance. However, the rate of increase varied according to the applied load.

In their study on AISI M2 steel, Li et al. [13] applied cryogenic treatment for 1, 4, 12, and 24 h (−120 °C) and reported that the highest hardness increase was obtained in the sample that was cryogenically treated for 24 h at the end of the study. Parcianello et al. [14] applied deep cryogenic treatment on AISI M2 tool steel for 20 and 28 h. The study reported that cryogenic treatment increased wear resistance and impact durability simultaneously. Higher wear resistance was achieved with the cryogenic treatment applied for 28 h. AISI H11 steel was subjected to cryogenic treatment by Katoch et al. [15] at two distinct temperatures (−154 °C and −184 °C) and for three different holding periods (6 h, 21 h, and 36 h). The sample that underwent a 6 h deep cryogenic treatment at −184 °C had the highest measured hardness, but a 36 h cryogenic treatment at −154 °C produced the highest measured toughness. The sample that underwent cryogenic treatment at −154 °C achieved the maximum tensile strength after 21 h of treatment. The tensile strength of other cryogenically treated samples was found to be lower than that of the untreated sample. The impact of cryogenic treatment on the mechanical and microstructural characteristics of AISI 430 stainless steels was investigated by Şenel et al. [16]. Following conventional heat treatment, the samples were stored for 12, 24, 36, and 48 h of deep cryogenic treatment at −140 °C. The samples that underwent cryogenically treated conditions for 24 h and 48 h, respectively, yielded the highest yield strength and tensile strength as a consequence of the research. Singh and Pandey [17] applied deep cryogenic treatment on Nimonic-90 steel for 24 and 48 h and found that up to 53% higher hardness was obtained after the deep cryogenic treatment applied for 48 h. Das et al. [18] employed cryogenic treatment at various holding durations (1, 12, 36, 60, 84, and 132 h) at −196 °C to ascertain the ideal holding period for AISI D2 steel that has undergone cryogenic treatment to provide maximal wear resistance. The study’s findings showed that samples that had a 36 h cryogenic treatment had the best wear resistance. Ti-6Al-4V alloy’s hardness and wear resistance were examined by Gu et al. [19] in relation to the effects of cryogenic treatment. In this investigation, 3, 48, and 72 h were dedicated to the deep cryogenic treatment. The sample subject to 72 h of cryogenic treatment achieved the greatest levels of hardness and wear resistance.

Although it is generally reported in the literature that more advanced mechanical properties are obtained with a longer cryogenic treatment holding time, there are also studies reporting that cryogenic treatment improves mechanical properties up to a certain period of time, but has no effect or causes a decrease in mechanical properties for longer periods. It turns out that different results are obtained depending on the type of steel used and the conditions for applying the cryogenic treatment. Tool steel, widely known as medium- to high-carbon or alloy steel, is used to manufacture tools, dies, or molds for cutting or shaping metal or non-metal materials due to its special characteristics of high hardness, wear resistance, and deformation resistance. Research and development of the tribological behavior of tool steels is of great significance [11,20,21,22]. While there are studies on the mechanical and microstructural behavior of X210CrW12 tool steel under different heat treatment conditions, no study investigates the effects of deep cryogenic treatment holding times. This study, conducted to close this gap in the literature, is believed to contribute significantly to the literature. This work examined the impact of holding periods and deep cryogenic treatment on the mechanical and microstructural characteristics of X210CrW12 steel. Following quenching treatment, deep cryogenic treatment was applied to the steel samples at −180 °C for 12 h, 18 h, 24 h, and 36 h, and then all samples were tempered at 300 °C for 2 h. Following these heat treatments, the steel samples were compared by subjecting them to hardness measurement, tensile tests, and wear tests. In addition, all samples were evaluated microstructurally, and the best cryogenic treatment holding time was determined for X210CrW12 steel to gain superior mechanical properties.

## 2. Material and Methods

**Figure 1:**
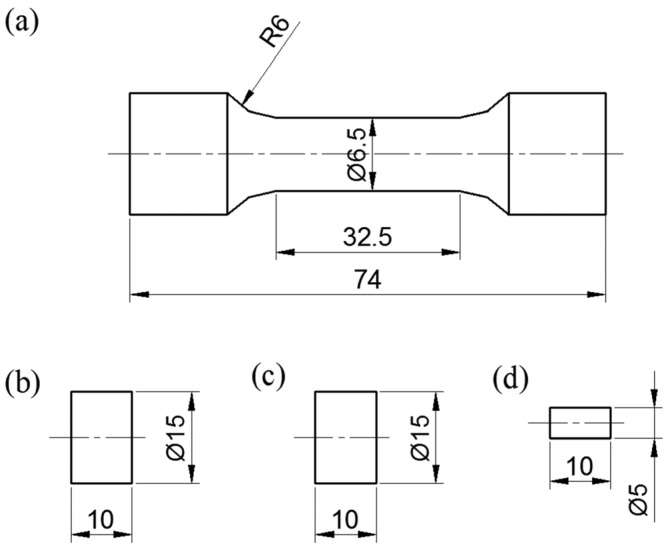
Dimensions of test samples (mm), (**a**) tensile test, (**b**) microstructural analysis, (**c**) hardness test, and (**d**) wear test.

**Figure 2:**
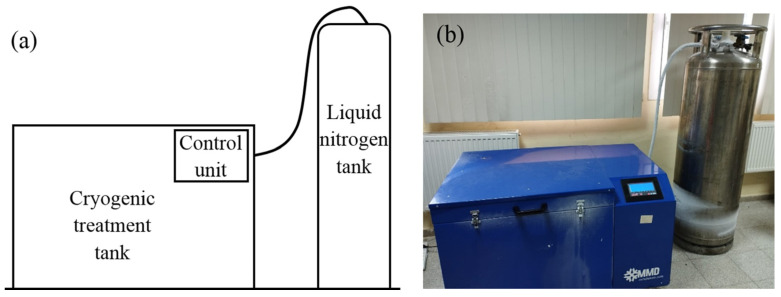
Cryogenic treatment setup: (**a**) schema of cryogenic setup; (**b**) photograph of cryogenic setup.

**Figure 3:**
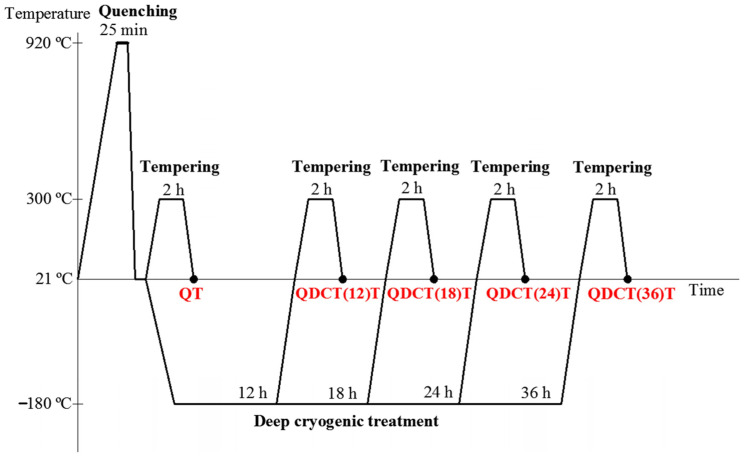
Details of heat treatments.

**Figure 4:**
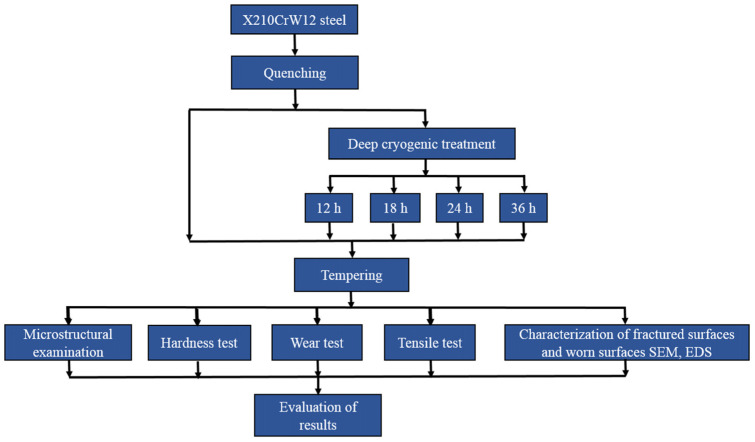
Overall methodology.

In this research, X210CrW12 steel, which is a cold work steel with a ledeburitic structure and 12% chromium, was used as the material. According to the spectral analysis result, the chemical composition of the steel is 2.28C-11.58Cr-0.14V-0.24Mo-0.4Mn-0.25Si-0.59W (w%). The dimensions of the samples prepared for microstructural and mechanical tests are shown in Figure 1. After being maintained at 920 °C for 25 min, X210CrW12 steel samples were quenched. After quenching, the steel samples were subjected to deep cryogenic treatment at −180 °C for 12, 18, 24, and 36 h using the device whose schematic view and photograph are given in Figure 2. A heating and cooling rate of 2 °C/min was used during heat treatment. Following deep cryogenic treatment, steel samples were exposed to a 2 h tempering heat treatment at 300 °C. The steel sample heat treatments are shown in Figure 3. Figure 4 illustrates the experimental methodology used in this work. The tests were performed independently on each steel sample, as the flow chart shows.

Samples were etched with Murakami solution (K_3_Fe(CN)_6_ and KOH) for microstructural investigation. A Quanta FEG 250 model (FEI, Hillsboro, OR, USA) scanning electron microscope was used to capture pictures of the microstructure, worn surfaces, and fracture surfaces. In addition, an image processing analysis program was used to determine the amount of carbide in steel samples subjected to different cryogenic treatments. Microstructure SEM images and SEM images after tensile tests were taken under an acceleration voltage of 20.00 kV, spot size of 3.0, magnification of 5000×, and SE mode. SEM images of worn surfaces were taken at 1000× magnification with other conditions being the same. X-ray diffraction (XRD) analysis was applied to determine the phase identification of the alloys using Cu/K-alpha1 radiation operated at a voltage of 40 kV and a current of 30 mA. Vickers microhardness measurements were determined on a Metkon Duroline M (Metkon Instruments, Gebze, Turkey) device by applying a 500 g load for 20 s. The samples’ Vickers hardness levels were ascertained by averaging five distinct measurements. Tensile tests were carried out in three repetitions with a 50 t Besmak device (Besmak, Istanbul, Turkey) according to ASTM E-8 standards [23]. Wear tests were performed according to the ASTM G99 standard [24]. A computer-controlled TRD Wear pin disk device was used to conduct the wear testing. During testing, the pin (i.e., specimen) was kept stationary and the circular disk was rotated. The pin material was X210CrW12, and the counter-face rotating disk was made of AISI 52100 (0.99C-0.35Mn-0.24Si-0.005S-0.009P-1.42Cr-Bal.Fe, wt.%). The disk specimen had a diameter of 100 mm and a thickness of 10 mm. The pin samples had a diameter of 5 mm and a length of 10 mm. The revolving disk’s counter face had a wear track diameter of 32 mm. The wear tests were performed with a 30 N force, at a speed of 0.75 m/s, and over a 900 m sliding distance. There was one way for the load to go, and no lubrication was utilized. The mass loss in samples determined the wear rate. No steps were taken prior to the samples being weighed. Equation (1) was used to obtain the wear rates (Ws) [25].

(1) $$\mathrm{Ws}=\frac{\Delta m}{L\times \rho \times F}$$

In this formula, Δm = mass loss (gr), L = distance of sliding (m), ρ = density (gr/mm^3^), and F = normal load (N).

## 3. Results and Discussion

**Figure 5:**
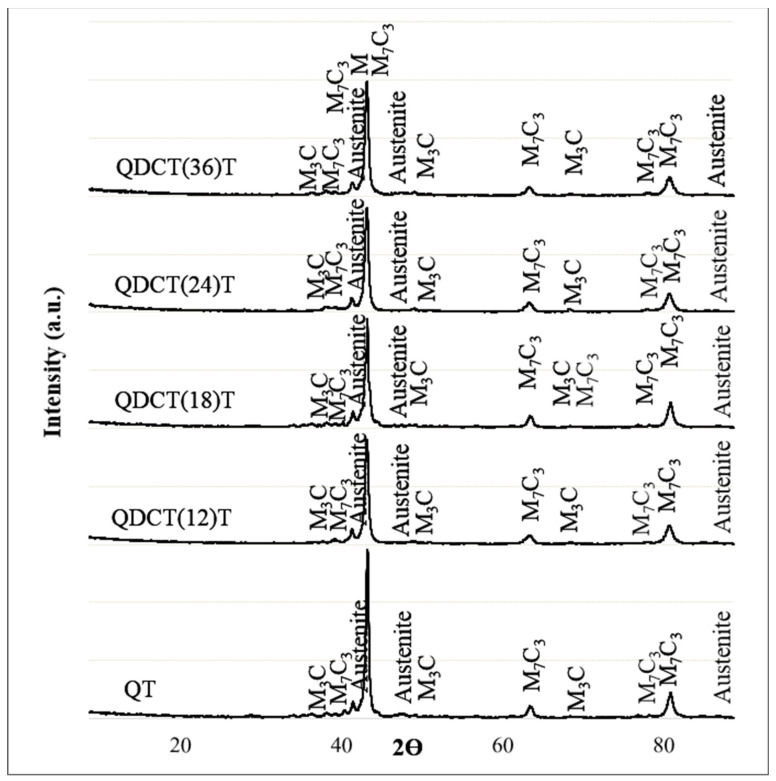
XRD analysis of heat-treated X210CrW12 steel samples.

The XRD analysis findings of the QT and QDCT(36)T steel samples are displayed in Figure 5. As shown in Figure 5, both heat treatments often result in phase peaks at the same 2ϴ degrees. The microstructure of steel samples exhibits the presence of M_3_C, M_7_C_3_, and austenite phases, as shown by XRD examination.

**Figure 6:**
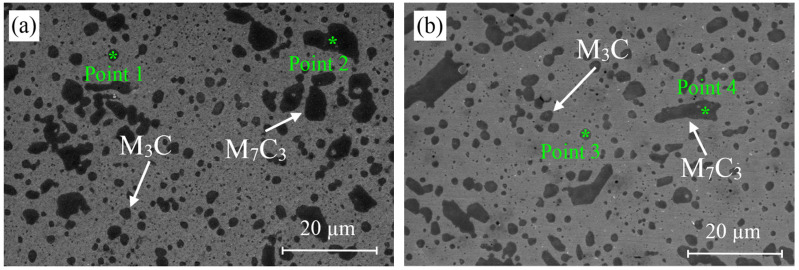

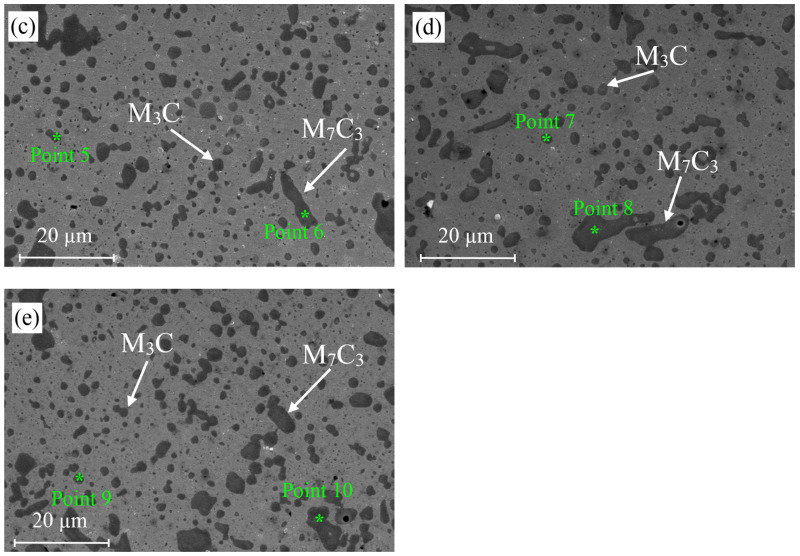
SEM images of different heat-treated X210CrW12 steel samples, (**a**) QT, (**b**) QDCT(12)T, (**c**) QDCT(18)T, (**d**) QDCT(24)T, and (**e**) QDCT(36)T.

**Figure 7:**
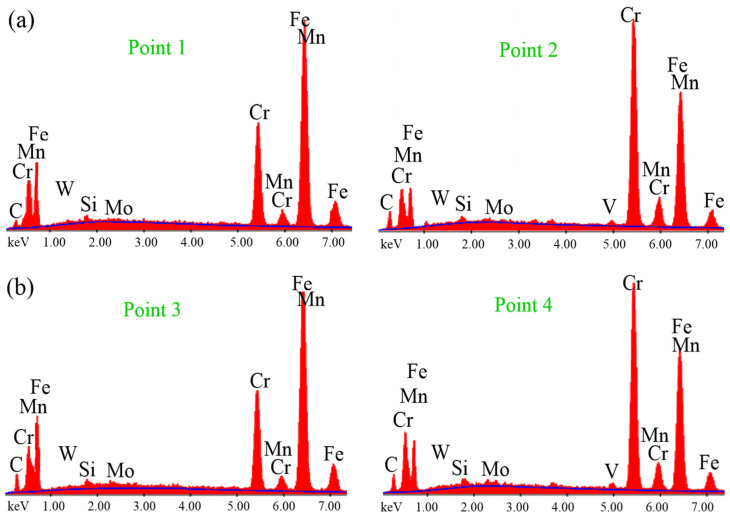

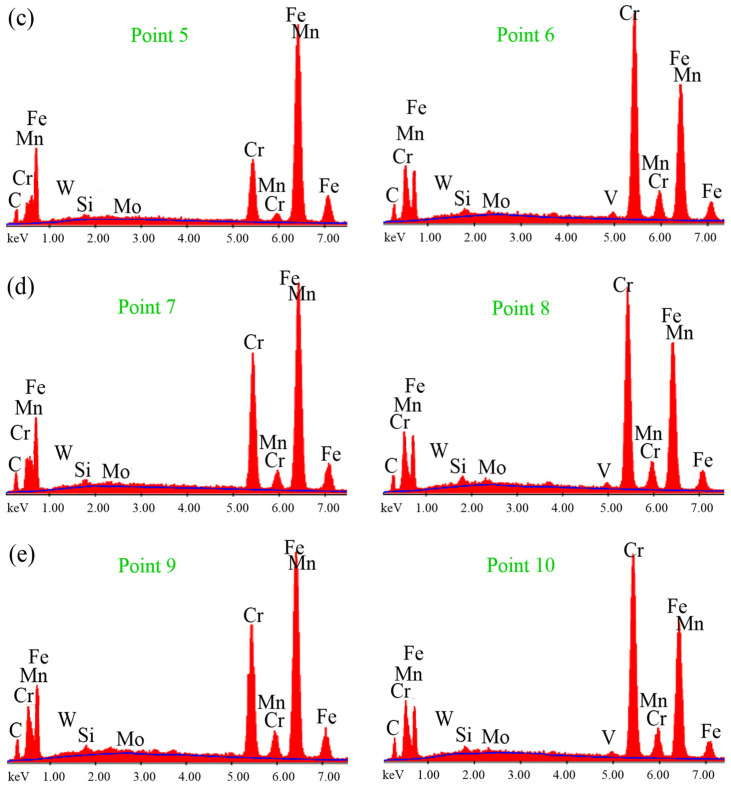
EDS analyses of different heat-treated X210CrW12 steel samples, (**a**) QT, (**b**) QDCT(12)T, (**c**) QDCT(18)T, (**d**) QDCT(24)T, and (**e**) QDCT(36)T.

**Figure 8:**
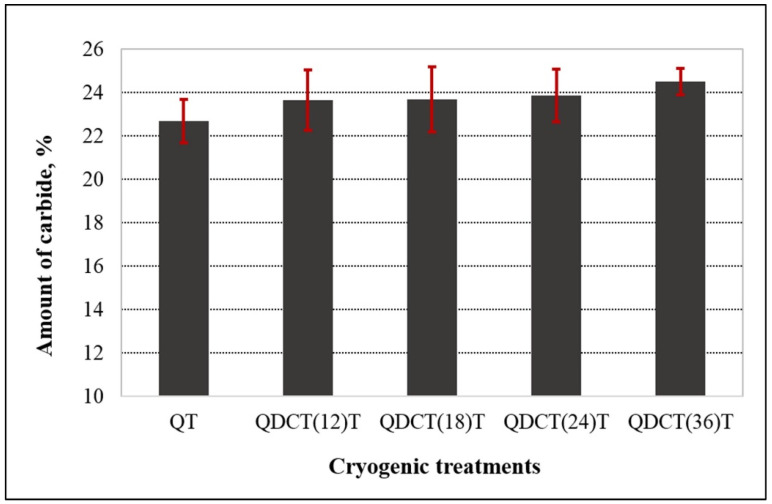
Graph of amount of carbide change of different heat-treated X210CrW12 steel samples.

Figure 6 shows the microstructure images of deep cryogenically treated steel samples at different holding times. In the microstructure images taken with SEM, the carbide grains in all samples are clearly visible in dark color. The first thing that stands out in the images is the microstructure with a more homogeneous carbide distribution after the cryogenic treatment. It is seen that carbide grains are more dense in certain regions in the microstructure of the untreated sample. Temperature decreases during cryogenic treatment, leading to lattice distortion and thermodynamic instability of martensite. Hence, carbon and alloying elements migrate to the nearby defects and segregate, forming fine carbides during tempering. The formation of high carbide contraction will increase wear resistance, reduce friction, and improve resistance [4,7]. Cryogenically treated samples had smaller sizes and higher carbide concentrations than untreated samples. Furthermore, all cryogenically treated samples had a more uniform carbide distribution. This indicates that the X210CrW12 steel undergoes carbide precipitation and a more uniform carbide dispersion as a result of the cryogenic treatment. M_7_C_3_ (large grains) and M_3_C (small grains) carbides are shown in the microstructure images. This was confirmed by EDS analyses (Figure 7). The M_3_C carbides in the SEM photos were identified by EDS analysis as having more Fe than Cr and a tiny amount of W and Mo. EDS analysis performed on the bigger carbides further validated the M_7_C_3_ carbide, which includes more Cr than Fe. Fe-rich carbide M_3_C (Points 1, 3, 5, 7, and 9) has an orthorhombic crystal structure. The crystal structure of M_7_C_3_ (Points 2, 4, 6, 8, and 10) is hexagonal. It is a Cr-rich carbide. It is seen in steels with only modest concentrations of other carbide-forming alloying elements and moderate to high Cr content [26]. The carbide amount of steel samples subjected to different cryogenic treatments was determined through the image processing analysis program, and the results are presented in Figure 8. Figure 8 reveals that the cryogenic process provided carbide precipitation in the microstructure of X210CrW12 steel. While the carbide amount of the QT sample is 22.66%, it is 23.63%, 23.66%, 23.85%, and 24.50% in the QDCT(12)T, QDCT(18)T, QDCT(24)T, and QDCT(36)T samples, respectively. A denser amount of carbide was achieved at higher deep cryogenic treatment holding times.

**Figure 9:**
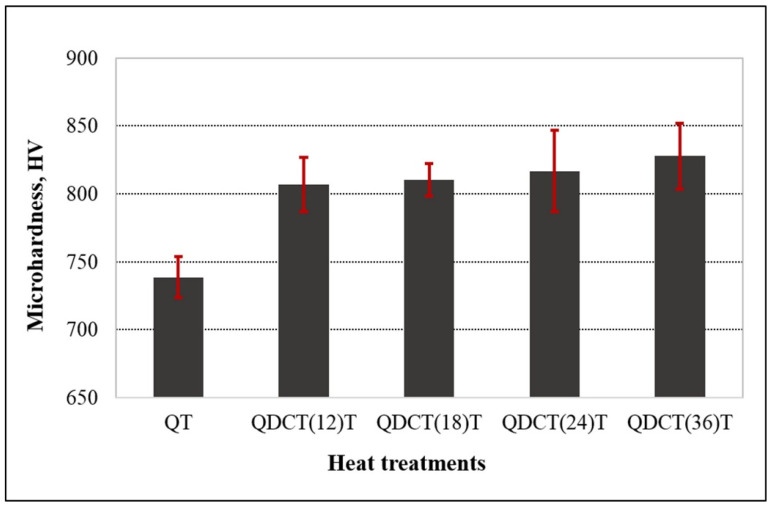
Microhardness change graph of different heat-treated X210CrW12 steel samples (500 g).

When the graph showing the microhardness measurement results is examined, it is seen that the deep cryogenic treatment increases the hardness of X210CrW12 steel (Figure 9). This increase in hardness in the steel samples is because the retained austenite phase was transformed into martensite by the cryogenic treatment [27]. The cryogenic treatment contributes to wear resistance due to fine η-carbide precipitation rather than the transformation of retained austenite to martensite [28]. This theory describes that using cryogenic treatment, coarse and randomly distributed carbide particles are refined into the most suitable form, called η-carbides, which improves the hardness and wear resistance without significantly affecting the toughness depending upon the material [29]. A dramatic increase in the population of η-carbides takes place during cryogenic treatment. These η-carbides precipitate in a size range up to 10 nm and are responsible for improving wear resistance in steel [4,30]. Microstructure images also revealed that the deep cryogenic treatment provided a denser amount of carbide and a more homogeneous carbide distribution in the steel samples. In the literature, in studies on 16Cr1Mo1Cu cast iron [31], TC4 titanium alloy [32], Nimonic-90 [17], EN52 Silchrome valve steel [33], Austempered Ductile Iron [34], 6061 aluminum alloy [35], and 18NiCrMo5 steel [36], cryogenic treatment was used. It has been shown that it has an effect on increasing hardness. The authors argued that this situation was achieved thanks to the change in the material’s microstructure with the cryogenic treatment.

While the hardness of the QT sample was 738.53 HV, it increased to 827.61 HV after deep cryogenic treatment. Compared to the QT sample, the hardness of the QDCT(12)T, QDCT(18)T, QDCT(24)T, and QDCT(36)T samples is higher by approximately 9.22%, 9.68%, 10.56%, and 12.06%, respectively. An increase in the hardness of the steel samples was observed with increasing cryogenic treatment holding time. A longer cryogenic treatment holding time resulted in a higher hardness increase. This situation is thought to be related to the increase in carbide density and more homogeneous carbide distribution as the cryogenic treatment holding time increases, as seen in the microstructural examination. The hardness values of the samples subjected to deep cryogenic treatment for 12, 18, and 24 h are close. In addition, an increase in the hardness values was observed with the increase in the cryogenic treatment waiting time, albeit slightly. The most significant increase among the cryogenic treatment samples was obtained in the sample subjected to deep cryogenic treatment for 36 h. The hardness of the QDCT(36)T sample is approximately 2.59% and 1.35% higher than the QDCT(12)T and QDCT(24)T samples. In the literature, Özbek and Özbek [37] found in their study that after cryogenic treatment, the carbide density in the microstructure of Sverker 21 steel increased with the increase in the cryogenic treatment holding time, and a more homogeneous carbide distribution was achieved. It has been observed that higher hardness is achieved by applying cryogenic treatment for longer. After deep cryogenic treatment was applied for 2, 18, and 36 h, a hardness increase of 11.75% was reported in 36 h. Singh and Pandey [17] applied deep cryogenic treatment on Nimonic-90 steel for 24 and 48 h and found that up to 53% higher hardness was acquired after the deep cryogenic treatment was applied for 48 h. Yıldız and Altan Özbek [38] applied deep cryogenic treatment on X17CrNi16-2 steel for 12, 18, and 36 h and achieved the highest hardness at 36 h, which is the longest holding time. Deep cryogenic treatment performed for 12 h produced more increased hardness, according to Kumar et al.’s [39] study, in which AISI D3 steel was subjected to the treatment for 12, 24, and 36 h. Amini et al. [40] applied deep cryogenic treatment on 1.2080 tool steel for 24, 36, 48, 72, 96, and 120 h and found that the hardness reached the maximum at the 36 h holding time. Hard rock cutting bits composed of tungsten carbide–cobalt (WC–Co) were subjected to deep cryogenic treatment by Chinnasamy et al. [41] at −196 °C for 12, 18, 24, 30, and 36 h. According to the deep cryogenic-treated wood’s mechanical characteristics, the hardness initially strengthened for up to 24 h (52.49%).

**Figure 10:**
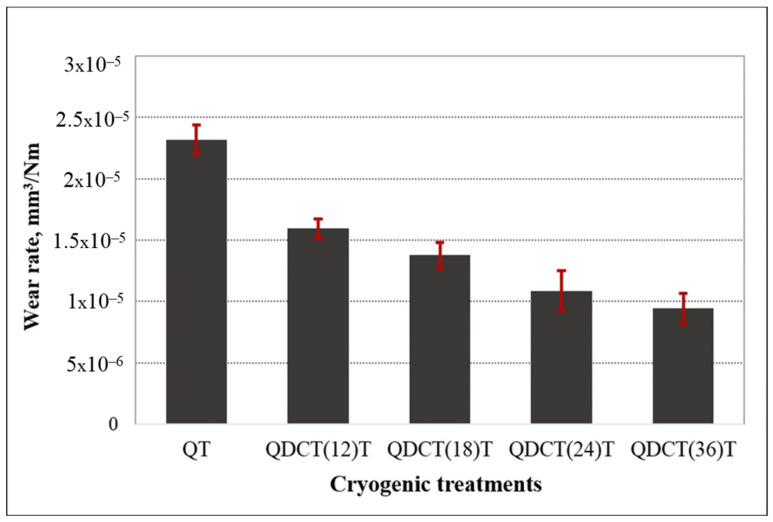
Different heat-treated X210CrW12 steel samples’ wear rate change graph.

When the graph showing the wear rates of steel samples as a result of the wear test is examined, it is seen that all samples with cryogenic treatment wear less than the untreated sample (Figure 10). This is explained by the cryogenic treatment’s ability to change the remaining austenite phase into martensite, which results in new carbide precipitation and a more uniform carbide distribution. Temperature decreases during cryogenic treatment, leading to lattice distortion and the thermodynamic instability of martensite. Hence, both carbon and alloying elements migrate to the nearby defects and segregate, forming fine carbides during tempering. The precipitated carbides reduce the internal tension of the martensite and minimize microcrack susceptibility, while the uniform distribution of fine carbides of high hardness enhances the wear resistance [4,7]. Generally, the harder a steel, the higher its wear resistance. The results presented in this study confirm this situation. AISI M2 tool steel underwent deep cryogenic treatment by Parcianello et al. [14]. According to the study, cryogenic treatment improved impact durability and wear resistance. Chen et al. [8] found that cryogenic treatment significantly increased the wear resistance of 51CrV4 steel. According to them, there was a 43.32% reduction in wear mass loss after the cryogenic treatment.

More wear resistance was produced by longer holding times for cryogenic treatment. Compared to the only quenched sample, the wear rate of the samples subjected to deep cryogenic treatment for 12 h, 18 h, 24 h, and 36 h is approximately 31.25%, 40.62%, 53.12%, and 59.37% lower, respectively. As seen in the microstructural examination, this situation is thought to be related to the increase in carbide density and more homogeneous carbide distribution with longer cryogenic treatment holding time. All these changes in the microstructure increase the hardness of the steel, which in turn increases wear resistance. It has been stated in the literature that higher carbide content may cause a decrease in wear resistance in some cases. Kumar et al. [42] emphasized in their study that when the abrasive Vibenite 480 (higher carbide content, less binder phase) was observed to break down, the carbide loss became more intense under dry erosion conditions at higher speeds, which caused a decrease in wear resistance. After a certain degree of increase in the carbide density in the material, it may tend to decrease and cause a third body effect or, if not, a burying effect, which can significantly change the wear mechanisms. However, in this study, the sample with a higher carbide content was less worn. In the literature, Li et al. [13] reported that the highest wear resistance after cryogenic treatment (−120 °C) applied to AISI M2 steel for 1, 4, 12, and 24 h was obtained in the sample that was cryogenically treated for 24 h. In another study, Özbek and Özbek [37] found that, after cryogenic treatment, the carbide density in the microstructure of Sverker 21 steel increased with the increase in the cryogenic treatment soaking time and a more homogeneous carbide distribution was achieved. It has been observed that higher hardness and wear resistance are obtained by applying cryogenic treatment for longer. After deep cryogenic treatment is applied to Sverker 21 steel for 2, 18, and 36 h, the wear rate is 64% lower in the more extended holding period of 36 h. In their study, Gu et al. [19] applied deep cryogenic treatment to Ti-6Al-4V alloy for three periods, 3 h, 48 h, and 72 h, and achieved the highest wear resistance in 72 h. Das et al. [18] carried out cryogenic treatment at −196 °C with various holding durations (1, 12, 36, 60, 84, and 132 h) to ascertain the ideal holding period for the highest level of wear resistance in AISI D2 steels treated cryogenically. The study’s findings showed that samples treated for 36 h at low temperatures produced the highest wear resistance.

**Figure 11:**
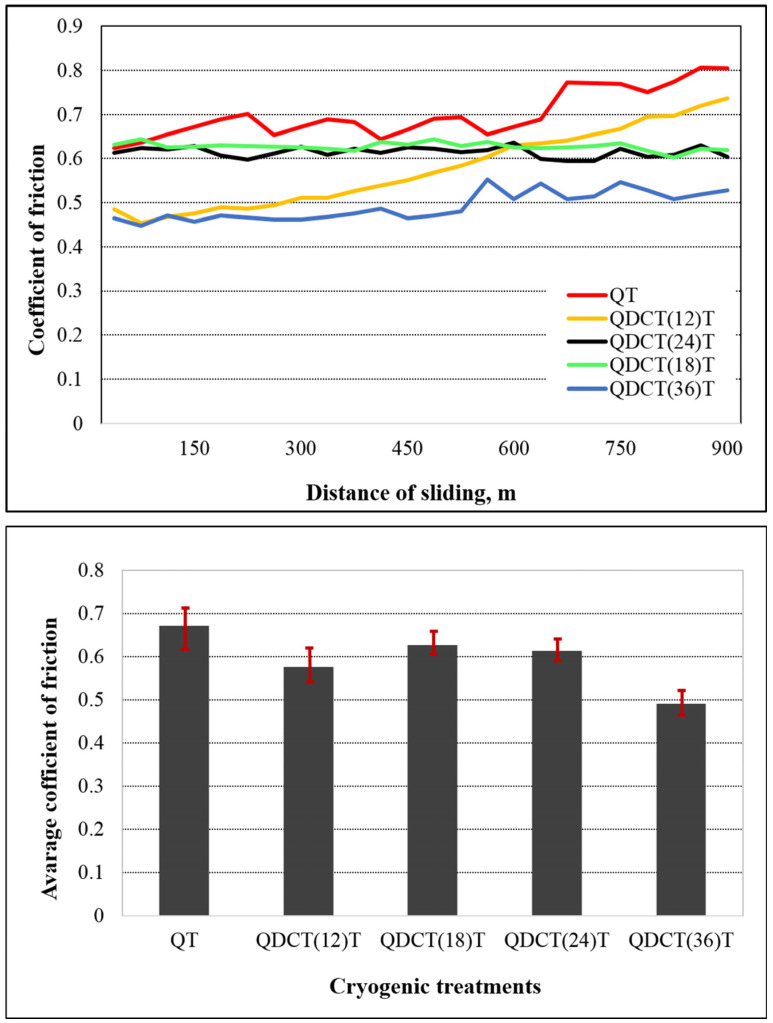
Different heat-treated X210CrW12 steel samples’ coefficient of friction change graph.

Figure 11 shows the change in the coefficient of friction (COF) sliding distance after cryogenic treatment. It is clear that the COF for the QT and QDCT(12)T samples exhibited significant fluctuations. However, with longer cryogenic treatment, the COF gradually stabilized, indicating an increase in the wear resistance of the steel sample to some extent. The periodic fluctuations in the COF can be attributed to the oxidation of the surface due to the heat generated during the friction process and the resulting formation of a smooth oxide film [43]. It was observed that the non-cryogenic treated (QT) sample gave the highest COF value, with an average friction coefficient value of 0.67. The lowest COF value was observed in the QDCT(36)T sample, with 0.49. After this sample, the lowest COF value was observed in the QDCT(12)T sample, which was 0.57. It was observed that the COF values of the QDCT(18)T and QDCT(24)T samples were quite close, at 0.62 and 0.61. In general, it is understood that cryogenic treatment causes a decrease in the friction coefficient.

**Figure 12:**
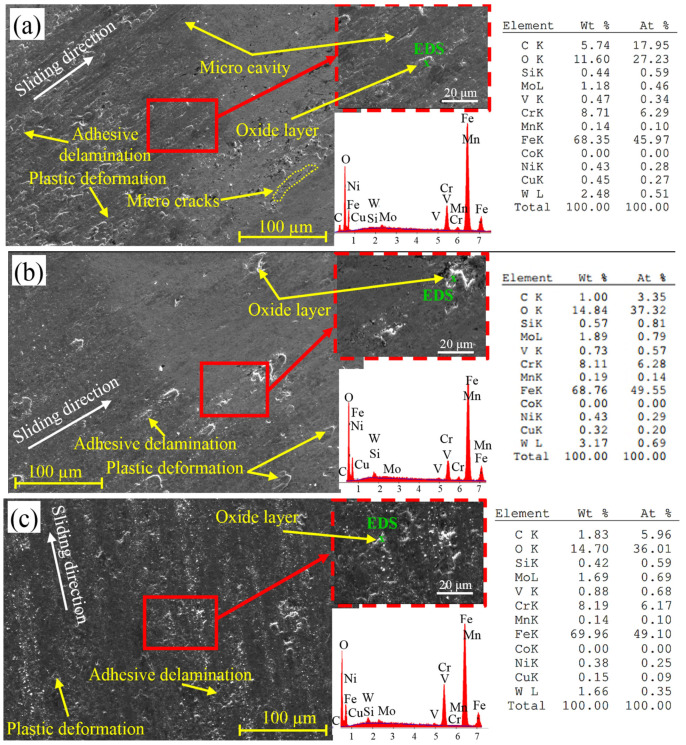

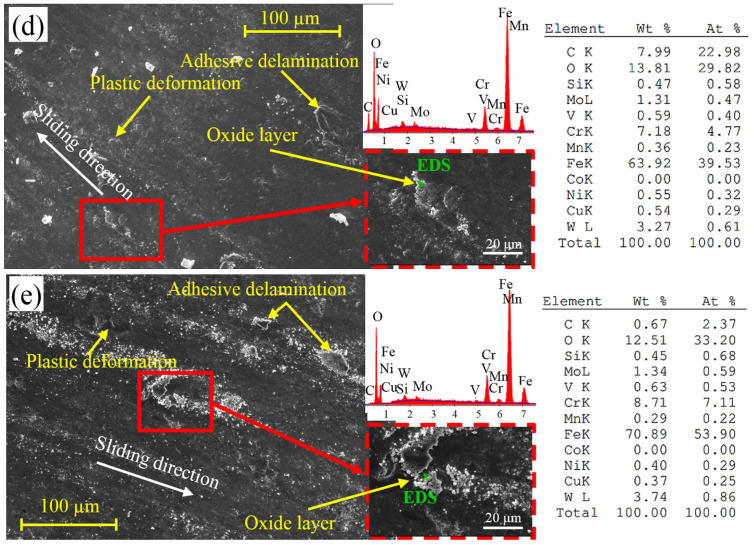
SEM pictures of several heat-treated X210CrW12 steel samples’ worn surfaces, (**a**) QT, (**b**) QDCT(12)T, (**c**) QDCT(18)T, (**d**) QDCT(24)T, and (**e**) QDCT(36)T.

**Figure 13:**
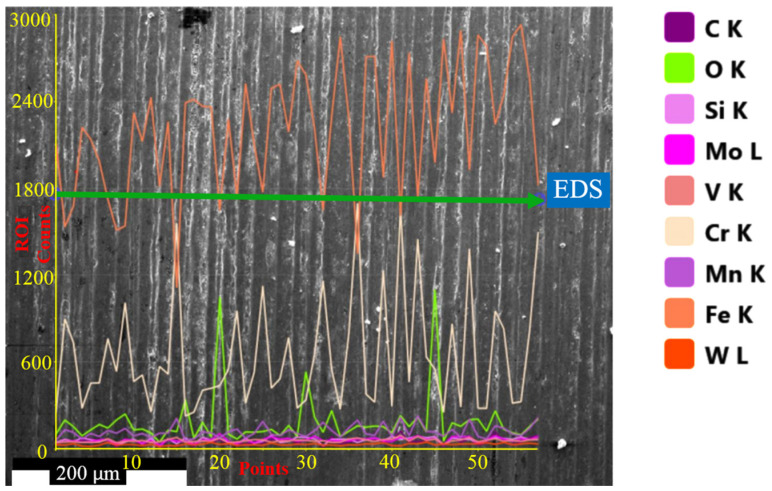
Linear EDS elemental analysis of the worn surface of the QT sample.

**Figure 14:**
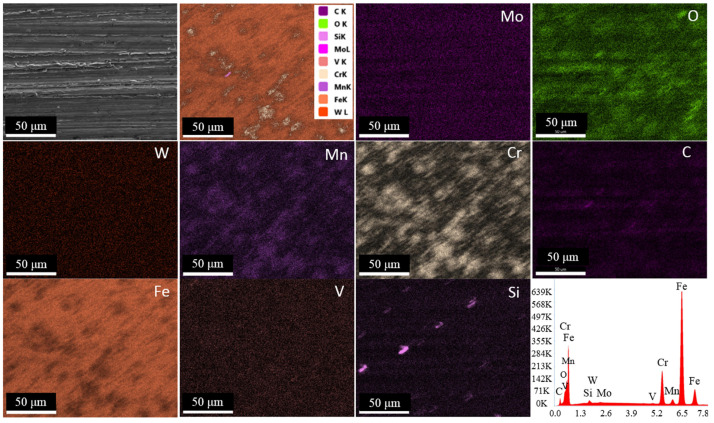
SEM image and EDS element maps of the worn surface of the QT sample.

**Figure 15:**
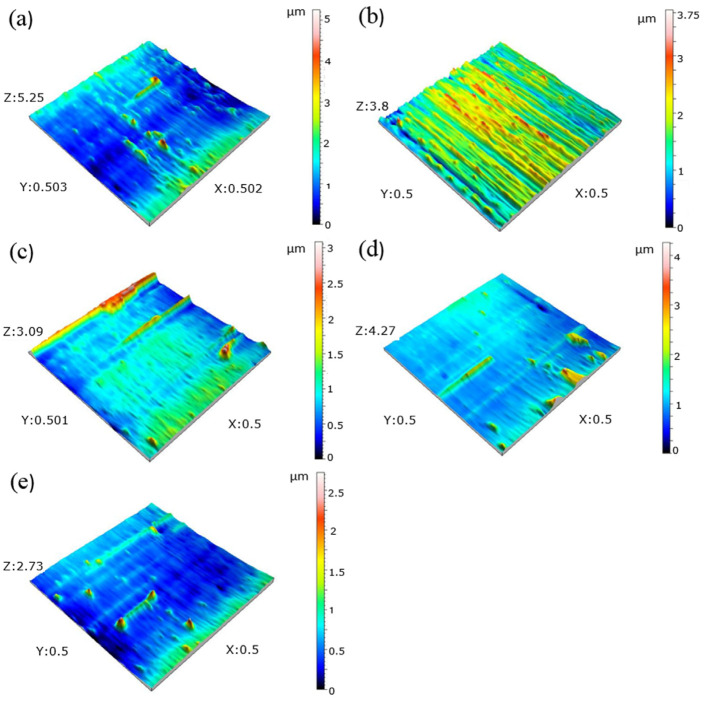
Three-dimensional optical profilometry images of worn surfaces, (**a**) QT, (**b**) QDCT(12)T, (**c**) QDCT(18)T, (**d**) QDCT(24)T, and (**e**) QDCT(36)T.

Following wear testing, the worn surfaces of the steel samples are depicted in SEM pictures in Figure 12. On the worn surfaces of every sample, layers of adhesive delamination and plastic deformation are visible. As the test sample slides across the disk, material flakes are drawn from the surface in the adhesive delamination process. All of the samples’ worn surfaces developed oxide layers over time. The oxide layer is thought to function as a protective layer, slowing down the rate of wear of the steel [44,45,46]. Kurşuncu [47] made a similar argument in his research, saying that wear resistance is positively impacted by oxidation on the worn surface. EDS analyses performed on the worn surfaces, shown in Figure 12, revealed the formation of oxide layers. The composition of the oxide layers includes Fe in amounts ranging from 63.92% to 70.89% and O in amounts ranging from 11.60% to 14.84%. In addition, linear EDS analysis (Figure 13) and EDS elemental mapping (Figure 14) performed on the worn surface of the QT sample also revealed the presence of oxide formations on the surface. On the other hand, EDS elemental maps also revealed the homogeneous distribution of elements on the surface. The SEM pictures demonstrate the formation of microcracks and microcavities in the QT sample. The early-stage fractures and voids will probably spread quickly, which will cause wear to develop even more. As a result of producing a more uniform and smaller carbide distribution, the cryogenic treatment appears to be crucial in strengthening the microstructure and inhibiting the formation of fractures. Furthermore, the transformation of leftover austenite into martensite inside the microstructure might be the cause of this behavior [28]. Figure 15 shows 3D optical profilometry images of worn surfaces. In the 3D images, wear marks and the depth of worn surfaces are clearly visible. The formation of an oxidation layer on the worn surfaces of the samples had a positive effect on wear. In general, the 3D images of the worn surfaces of all samples (except QDCT(12)T) exhibit similar characteristics. Clear peaks formed on the surfaces of all samples, but the highest peaks were in the sample without cryogenic treatment.

**Figure 16:**
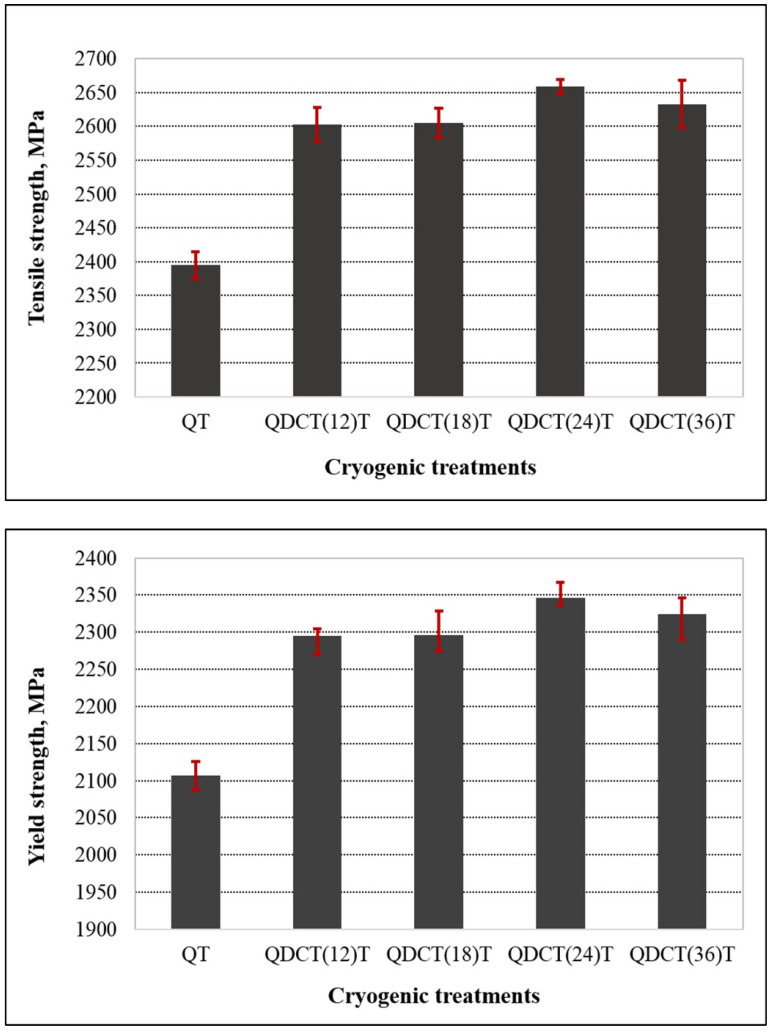
Tensile strength and yield strength change graphs of different heat-treated X210CrW12 steel samples.

The most often measured and reported parameter in tension test results is tensile strength. Tensile strength refers to the highest load ductile metals can support when subjected to severely limited uniaxial loading conditions. There is minimal correlation between this value and the structural members’ usable strength [48]. Figure 16 shows the change in tensile and yield strengths of steel samples subjected to deep cryogenic treatment for different periods. While the lowest tensile strength was seen in the QT sample, cryogenic treatment caused an increase of approximately 11% in the tensile strength and yield strength of X210CrW12 steel samples. Although the strength values of all deep cryogenically treated samples were higher than those of the QT sample, the tensile strength and yield strength reached their maximum with 24 h of cryogenic holding time.

When comparing cryogenic treatment of 815M17 steel to conventional heat treatment, Bensely et al. [48] revealed a little drop in tensile strength following cryogenic treatment; however, Sonia et al. [49] discovered that cryogenic treatment enhanced the tensile and yield strength of Al6082 aluminum alloy. Özbek [50] found that deep cryogenic treatment applied on Al 7050-T7451 alloy increased the tensile strength of the alloy by 17.36% to 43.23%. The cryogenic treatment applied for 24 h resulted in the greatest improvement in tensile strength among the deep cryogenic treatments carried out for 6 h, 12 h, 18 h, and 24 h. Yıldız and Altan Özbek [38] determined that cryogenic treatment applied for 12 h, 24 h, and 36 h increased the tensile strength of X17CRNI16-2 steel. They found that a higher tensile strength was obtained with an increase in cryogenic treatment holding time of up to 36 h. Kumar et al. [51] found that cryogenic treatment applied to a Bisphenol-based Polymer Composite for 24 and 72 h increased the tensile strength, and a higher tensile strength was obtained with a longer holding time.

**Figure 17:**
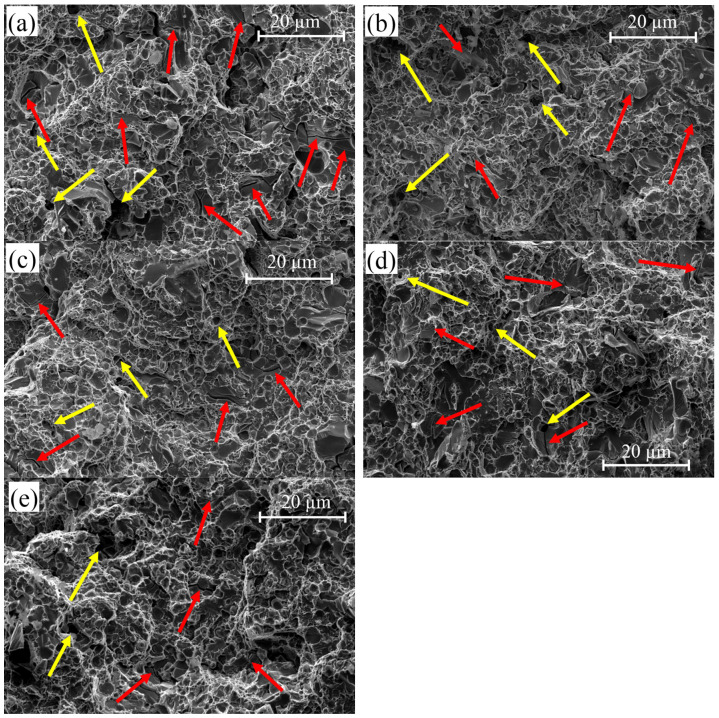
SEM images of the fracture surfaces after tensile tests of different heat-treated X210CrW12 steel samples (red arrows: intergranular and transgranular cracks; yellow arrows, small holes indicating ductile fracture), (**a**) QT, (**b**) QDCT(12)T, (**c**) QDCT(18)T, (**d**) QDCT(24)T, and (**e**) QDCT(36)T.

To analyze the mechanisms of the tensile property change of X210CrW12 steel after cryogenic treatment, the tensile fracture morphology was examined (Figure 17). The morphology of tensile fracture of all samples indicates that intergranular ductile fracture and transgranular ductile fracture occurred. Red arrows indicate intergranular and transgranular cracks. Intergranular ductile fracture occurs if microvoids form at the grain boundaries between grains. It is a high-energy fracture type. In transgranular ductile fracture, the bond between the voids within the grains becomes thinner and breaks, and the crack spreads. It is a high-energy fracture. The morphologies of the tensile fractures show the formation of small holes (indicated by yellow arrows), which also indicate ductile fracture. These surfaces have few small pits and many fracture planes and torn edges with local plastic deformation.

## 4. Conclusions

In this investigation, X210CrW12 steel underwent deep cryogenic treatment at −180 °C for 12, 18, 24, and 36 h following quenching. All samples were subsequently tempered for two hours at 300 °C. As a result of these processes, mechanical and microstructural changes of X210CrW12 steel were examined. The following conclusions can be drawn from the present work:

1. The investigation results showed that X210CrW12 steel had microstructural changes due to deep cryogenic treatment. Both new carbide precipitation and more uniform carbide dispersion were produced by deep cryogenic treatment. It was shown that after the deep cryogenic treatment was administered for 36 h, a more intense carbide content developed.
2. Compared to the quenched sample, the hardness of X210CrW12 steel increased during deep cryogenic treatment, ranging from 9.22% to 12.06%. The sample that underwent a 36 h deep cryogenic treatment had the maximum hardness.
3. The wear tests revealed that the deep cryogenic treatment improved X210CrW12 steel’s wear resistance. There was less wear on the sample that was subjected to a lengthier deep cryogenic treatment. The sample that underwent a 36 h deep cryogenic treatment had a wear rate of 59.27% less than the quenched sample.
4. Plastic deformation and adhesive delamination layers developed on the steel samples’ worn surfaces. Furthermore, oxide coatings formed on all of the samples’ worn surfaces over time. The QT sample developed microcracks and microvoids as a result of the wear test.
5. Deep cryogenic treatment increased the yield and tensile strength values of X210CrW12 steel at varying rates of up to 11%. The highest tensile strength and yield strength were obtained in samples subjected to deep cryogenic treatment for 24 h.

## Data Availability

The original contributions presented in this study are included in the article. Further inquiries can be directed to the corresponding author.

## References

[1] Zhirafar S., Rezaeian A., Pugh M. (2007). Effect of Cryogenic Treatment on the Mechanical Properties of 4340 Steel. J. Mater. Process. Technol..

[2] Bensely A., Prabhakaran A., Mohan Lal D., Nagarajan G. (2005). Enhancing the Wear Resistance of Case Carburized Steel (En 353) by Cryogenic Treatment. Cryogenics.

[3] Özbek N.A., Çiçek A., Gülesin M., Özbek O. (2014). Investigation of the Effects of Cryogenic Treatment Applied at Different Holding Times to Cemented Carbide Inserts on Tool Wear. Int. J. Mach. Tools Manuf..

[4] Kalsi N.S., Sehgal R., Sharma V.S. (2010). Cryogenic Treatment of Tool Materials: A Review. Mater. Manuf. Process..

[5] Çetin A., Çiçek A. Cryogenic Treatment for Tool Steels. Proceedings of the 4th International Symposium on Innovative Technologies in Engineering and Science.

[6] Tyshchenko A.I., Theisen W., Oppenkowski A., Siebert S., Razumov O.N., Skoblik A.P., Sirosh V.A., Petrov Y.N., Gavriljuk V.G. (2010). Low-Temperature Martensitic Transformation and Deep Cryogenic Treatment of a Tool Steel. Mater. Sci. Eng. A.

[7] Huang J.Y., Zhu Y.T., Liao X.Z., Beyerlein I.J., Bourke M.A., Mitchell T.E. (2003). Microstructure of Cryogenic Treated M2 Tool Steel. Mater. Sci. Eng. A.

[8] Chen Z., Jing L., Gao Y., Huang Y., Guo J., Yan X. (2023). Impact of Cryogenic Treatment Process on the Performance of 51CrV4 Steel. Materials.

[9] Darwin J.D., Mohan Lal D., Nagarajan G. (2008). Optimization of Cryogenic Treatment to Maximize the Wear Resistance of 18% Cr Martensitic Stainless Steel by Taguchi Method. J. Mater. Process. Technol..

[10] Yan X.G., Li D.Y. (2013). Effects of the Sub-Zero Treatment Condition on Microstructure, Mechanical Behavior and Wear Resistance of W9Mo3Cr4V High Speed Steel. Wear.

[11] Studeny Z., Krbata M., Dobrocky D., Eckert M., Ciger R., Kohutiar M., Mikus P. (2022). Analysis of Tribological Properties of Powdered Tool Steels M390 and M398 in Contact with Al_2_O_3_. Materials.

[12] Das D., Dutta A.K., Toppo V., Ray K.K. (2007). Effect of Deep Cryogenic Treatment on the Carbide Precipitation and Tribological Behavior of D2 Steel. Mater. Manuf. Process..

[13] Li J., Yan X., Liang X., Guo H., Li D.Y. (2017). Influence of Different Cryogenic Treatments on High-Temperature Wear Behavior of M2 Steel. Wear.

[14] Parcianello C.T., Fantineli D.G., Rosendo T.S., Reguly A., Tier M.A.D. (2023). Influence of the Heat Treatment on the Mechanical and Tribological Properties of Cryogenically Treated AISI M2 Steel. J. Mater. Res. Technol..

[15] Katoch S., Singh V., Sehgal R. (2019). Mechanical Properties and Microstructure Evaluation of Differently Cryogenically Treated AISI-H11 Steel. Int. J. Steel Struct..

[16] Şenel S., Koçar O., Kocaman E., Özdamar O. (2021). Investigation of the Mechanical and Microstructural Properties of AISI 430 Steels After Deep Cryogenic Treatment. Eur. J. Sci. Technol..

[17] Singh G., Pandey K.N. (2024). Effect of Deep Cryogenic Treatment, Tempering Temperature and Time on Hardness of Nimonic-90. Proc. Inst. Mech. Eng. Part E J. Process Mech. Eng..

[18] Das D., Dutta A.K., Ray K.K. (2009). Optimization of the Duration of Cryogenic Processing to Maximize Wear Resistance of AISI D2 Steel. Cryogenics.

[19] Gu K., Wang J., Zhou Y. (2014). Effect of Cryogenic Treatment on Wear Resistance of Ti-6Al-4V Alloy for Biomedical Applications. J. Mech. Behav. Biomed. Mater..

[20] Ekaputra R.C. (2023). The Effect of Parameters in Cryogenic Treatment on Mechanical Properties of Tool Steel: A Review. J. Mater. Explor. Find..

[21] Essam M.A., Shash A.Y., El-Fawakhry M.K., El-Kashif E., Megahed H. (2023). Effect of Deep Cryogenic Treatment on Wear Behavior of Cold Work Tool Steel. Metals.

[22] Krbata M., Eckert M., Bartosova L., Barenyi I., Majerik J., Mikuš P., Rendkova P. (2020). Dry Sliding Friction of Tool Steels and Their Comparison of Wear in Contact with ZrO2 and X46Cr13. Materials.

[23] (2016). Standard Test Methods for Tension Testing of Metallic Materials (E8/E8M).

[24] (2017). Standard Test Method for Wear Testing with a Pin-on-Disk Apparatus.

[25] Chin C.W., Yousif B.F. (2009). Potential of Kenaf Fibres as Reinforcement for Tribological Applications. Wear.

[26] Vander G.F. (2004). Metallographic Techniques for Tool Steels. ASM Handbook: Metallography and Microstructures.

[27] Kara F., Özbek O., Altan Özbek N., Uygur İ. (2021). Investigation of the Effect of Deep Cryogenic Process on Residual Stress and Residual Austenite. Gazi J. Eng. Sci..

[28] Yang H.S., Jun W., Bao-Luo S., Hao-Huai L., Sheng-Ji G., Si-Jiu H. (2006). Effect of Cryogenic Treatment on the Matrix Structure and Abrasion Resistance of White Cast Iron Subjected to Destabilization Treatment. Wear.

[29] Vadivel K., Rudramoorthy R. (2009). Performance Analysis of Cryogenically Treated Coated Carbide Inserts. Int. J. Adv. Manuf. Technol..

[30] Meng F., Tagashira K., Azuma R., Sohma H. (1994). Role of Eta-Carbide Precipitations in the Wear Resistance Improvements of Fe-12Cr-Mo-V-1.4C Tool Steel by Cryogenic Treatment. ISIJ Int..

[31] Wang J., Xiong J., Fan H., Yang H.S., Liu H.H., Shen B.L. (2009). Effects of High Temperature and Cryogenic Treatment on the Microstructure and Abrasion Resistance of a High Chromium Cast Iron. J. Mater. Process. Technol..

[32] Lan X., Xu Y., Li J., Gong Y., Shi M. (2024). The Influence of Deep Cryogenic Treatment (DCT) on the Microstructure Evolution and Mechanical Properties of TC4 Titanium Alloy. Materials.

[33] Saranraj I., Ganesan S., Čepová L., Elangovan M., Beránek L. (2022). Microstructure, Mechanical and Wear Behaviour of Deep Cryogenically Treated EN 52 Silchrome Valve Steel. Materials.

[34] Çakir F.H. (2022). The Effect of Cryogenic Treatment on Hardness, Toughness, and Tribological Properties of Austempered Ductile Iron with Different Nickel Contents. Int. J. Met..

[35] Niu X., Chen Z., Jing L., Huang Y., Liu Y. (2024). Effect of Cryogenic Treatment on Residual Stress and Microstructure of 6061 Aluminum Alloy and Optimization of Parameters. Materials.

[36] Baldissera P., Delprete C. (2009). Effects of Deep Cryogenic Treatment on Static Mechanical Properties of 18NiCrMo5 Carburized Steel. Mater. Des..

[37] Altan Özbek N., Özbek O. (2022). Effect of Cryogenic Treatment Holding Time on Mechanical and Microstructural Properties of Sverker 21 Steel. Mater. Test..

[38] Yildiz E., Ozbek N.A. (2023). Investigation of the Effects of Deep Cryogenic Treatment on the Microstructure, Hardness, Strength and Wear Resistance of X17CrNi16-2 Martensitic Stainless Steel. Surf. Rev. Lett..

[39] Kumar S., Nagaraj M., Arunkumar B., Khedkar N.K. (2019). Effect of Deep Cryogenic Treatment on the Mechanical Properties of AISI D3 Tool Steel. Int. J. Mater. Eng. Innov..

[40] Amini K., Akhbarizadeh A., Javadpour S. (2012). Investigating the Effect of Holding Duration on the Microstructure of 1.2080 Tool Steel during the Deep Cryogenic Heat Treatment. Vacuum.

[41] Chinnasamy M., Rathanasamy R., Palaniappan S.K., Pal S.K., Muthuswamy P., Korrayi R.R., Uddin M.E. (2023). Microstructural and Tribological Characterization of Cryogenic Treated WC-Co Cutting Bits under Different Holding Times for Rock Cutting Applications. J. Mater. Eng. Perform..

[42] Kumar R., Antonov M., Beste U., Goljandin D. (2020). Assessment of 3D Printed Steels and Composites Intended for Wear Applications in Abrasive, Dry or Slurry Erosive Conditions. Int. J. Refract. Met. Hard Mater..

[43] Du Y., Yuan X., Hu H., Tian P., Long M., Chen D. (2024). Microstructure Evolution and Wear Resistance Enhancement of Nano-NiCoC Alloy Coatings via Cryogenic Treatment. Surf. Coat. Technol..

[44] Prince R.M.R., Selvakumar N., Arulkirubakaran D., Singh S.C.E., Ramkumar T., Kumar R.M. (2020). Surface Structural Features and Wear Analysis of a Multilayer Ti6Al4V-B_4_C Thin Film Coated AISI 1040 Steel. Mater. Res. Express.

[45] Kumar R., Hussainova I., Antonov M., Maurya H.S., Rodríguez Ripoll M. (2024). Temperature-Induced Wear Micro-Mechanism Transition in Additively Deposited Nickel Alloys with Different Solid Lubricants. Wear.

[46] Escherová J., Krbata M., Kohutiar M., Barényi I., Chochlíková H., Eckert M., Jus M., Majerský J., Janík R., Dubcová P. (2024). The Influence of Q & T Heat Treatment on the Change of Tribological Properties of Powder Tool Steels ASP2017, ASP2055 and Their Comparison with Steel X153CrMoV12. Materials.

[47] Kurşuncu B. (2021). The Effect of Cryogenic Treatment on Dry Sliding Wear Mechanisms in Hard Coatings. Ind. Lubr. Tribol..

[48] Bensely A., Senthilkumar D., Mohan Lal D., Nagarajan G., Rajadurai A. (2007). Effect of Cryogenic Treatment on Tensile Behavior of Case Carburized Steel-815M17. Mater. Charact..

[49] Sonia P., Verma V., Saxena K.K., Kishore N., Rana R.S. (2019). Effect of Cryogenic Treatment on Mechanical Properties and Microstructure of Aluminium 6082 Alloy. Mater. Today Proc..

[50] Özbek O. (2023). Effects of Deep Cryogenic Treatment with Different Holding Times on the Mechanical Properties of Al 7050-T7451 Alloy Friction Stir Welding. Mater. Test..

[51] Shashi Kumar M.E., Mohan Kumar S., Ravi Kumar V. (2019). Effect of Cryogenic Treatment on Bisphenol Based Polymer Composite on Mechanical Properties. Int. J. Recent Technol. Eng..

